# Identification and validation of potential prognostic and predictive miRNAs of epithelial ovarian cancer

**DOI:** 10.1371/journal.pone.0207319

**Published:** 2018-11-26

**Authors:** Kira Philipsen Prahm, Claus Høgdall, Mona Aarenstrup Karlsen, Ib Jarle Christensen, Guy Wayne Novotny, Estrid Høgdall

**Affiliations:** 1 Department of Pathology, Molecular unit, Danish CancerBiobank, Herlev University Hospital, Herlev, Denmark; 2 Gynecological Clinic, The Juliane Marie Center, Rigshospitalet, Copenhagen University Hospital, Copenhagen, Denmark; University of South Alabama Mitchell Cancer Institute, UNITED STATES

## Abstract

**Background:**

Ovarian cancer is the leading cause of death by gynecologic cancers in the Western world. The aim of the study was to identify microRNAs (miRNAs) associated with prognosis and/or resistance to chemotherapy among patients with epithelial ovarian cancer.

**Methods:**

Using information from the Pelvic Mass Study we identified a cohort of women with epithelial ovarian cancer. Tumor tissues were then collected and analyzed by global miRNA microarrays. MiRNA profiling was then linked to survival and time to progression using Cox proportional-hazards regression models. Logistic regression models were used for the analysis of resistance to chemotherapy. Our results were validated using external datasets retrieved from the NCBI Gene Expression Omnibus database.

**Results:**

A total of 197 patients with epithelial ovarian cancer were included for miRNA microarray analysis. In multivariate analyses we identified a number of miRNAs significantly correlated with overall survival (miR-1183 (HR: 1.42, 95% CI:1.17–1.74, *p* = 0.0005), miR-126-3p (HR: 1.38, 95% CI:1.11–1.71, *p =* 0.0036), time to progression (miR-139-3p (HR: 1.48, 95% CI: 1.13–1.94, *p =* 0.0047), miR-802 (HR: 0.48, 95% CI: 0.29–0.78, *p* = 0.0035)), progression free survival (miR-23a-5p (HR:1.32, 95% CI:1.09–1.61, *p =* 0.004), miR-23a-3p (HR:1.70, 95% CI:1.15–2.51, *p =* 0.0074), miR-802 (HR: 0.48, 95% CI: 0.29–0.80, *p* = 0.0048)), and resistance to chemotherapy (miR-1234 (HR: 0.26, 95% CI: 0.11–0.64, *p* = 0.003)). A few miRNAs identified in our training cohort, were validated in external cohorts with similar results.

**Conclusion:**

Eight miRNAs were identified as significant predictors of overall survival, progression free survival, time to progression, and chemotherapy resistance. A number of these miRNAs were significantly validated using external datasets. Inter-platform and inter-laboratory variations may have influence on the ability to compare and reproduce miRNA results. The use of miRNAs as potential markers of relapse and survival in ovarian cancer warrants further investigation.

## Introduction

Ovarian cancer (OC) is the most lethal gynecologic malignancy in the Western world [1]. It affects approximately 500 women annually and is the cause of close to 400 deaths per year in Denmark [2, 3]. The high mortality is, among other factors, a consequence of advanced disease stage at the time of diagnosis; approximately 70% of patients with OC are diagnosed at advanced stages (FIGO stage III-IV) where the 5-year survival rate is less than 30% [1, 4, 5]. The standard primary treatment for OC patients includes primary debulking surgery followed by adjuvant, platinum-based chemotherapy. Despite an initial good response to chemotherapy, where clinical remission is achieved, the majority of patients eventually experience relapses [6]. Patients with recurrent disease are commonly offered an alternative chemotherapy regimen, however the response rates at this point is much lower [7]. Today, the only clinically implemented biomarker used for OC monitoring is cancer antigen 125 (CA125). However, the sensitivity and specificity are known to be poor for prediction of early relapse and CA125 cannot be used in prediction of response to chemotherapy [8]. Therefore, there is a need for methods or biomarkers that can identify patients at risk of early relapse and help allocate patients to the most optimal chemotherapy, hereby eventually improving survival.

MicroRNAs (miRNA) are a large class of small non-coding RNA-molecules that, within the last decade, have shown to be potential biomarkers for cancer [9]. miRNAs regulate gene expression by imperfect binding to complementary sites in the 3’ untranslated regions of messenger RNAs, and have shown to regulate important cellular functions such as cell growth, differentiation and apoptosis [10, 11]. Numerous studies have shown miRNAs to be aberrantly expressed in human cancers and demonstrated that miRNAs can act as both tumor-suppressor genes and oncogenes [10, 12, 13]. Several miRNAs have also been identified as potential biomarkers for survival outcome and response to chemotherapy in OC. However there have been conflicting results regarding the function of individual miRNAs, and the full understanding of their function in OC still needs further elucidation before they can be tested in clinical trials [9].

In the current study, we aimed to identify and validate single miRNAs prognostic and predictive of overall survival (OS), time to progression (TTP), progression free survival (PFS), and chemotherapy resistance. To demonstrate the validity of our data we tested the performance of 35 miRNAs included in a previously identified miRNA-based predictor (MiROvaR) of early relapse and progression [14]. We then aimed to validate the identified miRNAs of significance in our analyses, on external cohorts.

## Material and methods

### Explorative cohort

#### Patients and tissue

Patients recruited for our training cohort were included from the Pelvic Mass study, elaborated in previous papers [15, 16]. The Pelvic Mass study is a prospective ongoing clinical study initiated in 2004 at the Gynecological Clinic, Rigshospitalet, Copenhagen University Hospital, Denmark, with the overall purpose to identify diagnostic and prognostic factors for OC [17]. Clinical information on all patients is continuously updated in the Danish Gynecologic Cancer Database and tumor tissue is handled and stored by the Danish CancerBiobank [5, 16]. Inclusion criteria for the current study were: Primary debulking surgery and EOC confirmed by a gynecologic pathologist. Exclusion criteria were: Previous or another concomitant cancer or insufficient tissue material for analysis.

UL/CT/MRI/PET-CT scans, serum CA125, and patient symptoms were used for evaluation of relapse or progression of disease. In a few cases (n = 18) no information of relapse or progression of disease were registered, but in these cases, start of second-line chemotherapy was considered as time of relapse or progressive disease. TTP was calculated as the time elapsed from end of first line chemotherapy until progressive disease, recurrent disease, start of second line chemotherapy or death, which ever occurred first. Progression free survival (PFS) was defined as time from primary surgery until progression of or recurrent disease, death, or last date of follow-up for patients still alive. Patients who experienced progressive disease during first-line chemotherapy, or those who suffered from recurrent disease within the first six months after end of first-line chemotherapy, were considered chemotherapy resistant. Patients who experienced progressive disease during treatment, or within the first four weeks after end of chemotherapy, were considered chemotherapy refractory. Patients without progressive or recurrent disease, or cases where progressive or recurrent disease occurred more than six months after end of first-line chemotherapy, were considered chemotherapy sensitive. OS was calculated as the time from date of surgery until death of any cause, or until January 17, 2015, which ever came first. Surgical outcome was defined as complete cytoreduction (no macroscopic visual tumor), optimal cytoreduction (tumor < 1cm) or suboptimal cytoreduction (tumor >1 cm). Suboptimal cytoreduction was further divided in tumor size > 1 cm and ≤2 cm or tumor size > 2 cm (Table 1). Only patients, who had received a minimum of two series of chemotherapy, were used for prediction of TTP and chemotherapy resistance.

**Table 1:** Baseline characteristics of 198 patients with EOC.

Median age in year (range)	64 (31–89)
Median OS in months	47.9 (95% CI: 39.8–56.0)
Histology	
Serous adenocarcinoma	162 (82%)
Mucinous adenocarcinoma	11 (6%)
Endometrioid adenocarcinoma	15 (8%)
Clear Cell adenocarcinoma	9 (5%)
FIGO stage	
I	31 (16%)
II	21 (11%)
III	119 (60%)
IV	26 (13%)
Histologic grade	
1	20 (10%)
2	102 (52%)
3	74 (38%)
Unknown	1 (<1%)
Residual tumor after surgery	
0	94 (48%)
<1 cm	32 (16%)
>1 cm ≤ 2 cm	22 (11%)
>2 cm	49 (25%)
Chemotherapy (n = 170)	
Carboplatin + Docetaxel	163 (96%)
Carboplatin + Paclitaxel	1 (<1%)
Carboplatin	5 (3%)
Chemotherapy-resistance	
>6 months (sensitive)	124 (73%)
<6 months (resistant)	26 (15%)
Chemotherapy-refractory	20 (12%)
Median follow-up (months)	88 (61–126)

EOC = epithelial ovarian cancer, OS = overall survival

FIGO = International Federation of Gynecology and Obstetrics

Clinical data for the current study are provided in S1 Clinical Data.

#### RNA purification and microarray analysis

Total RNA was extracted from formalin-fixed, paraffin-embedded (FFPE) tumor tissue. The RecoverAll (Ambion, Inc 2130 Woodward St. Austin, TX) total nuclei acid isolation kit for FFPE was used for the RNA extraction. Briefly, a tumor tissue section of 20μm, where tumor cell content was more than 50%, was used from each patient. The FFPE tumor tissue was deparaffinized in xylene, then washed in an alcohol solution to remove the xylene and subsequently digested with a protease to remove covalent bonds between RNA, DNA and other proteins. The RNA was then extracted on a glass-fiber filter, washed in high ethanol concentrations, and eluted. The quantity of total RNA was measured with a NanoDrop ND-1000 Spectrophotometer (NanoDrop Technology, Wilmington, Del). MiRNA was then labeled with biotin using FlashTag HSR Biotin RNA Labeling Kit (Affymetrix, Santa Clara, CA), and hybridized to GeneChip 1.0 miRNA microarrays (Affymetrix, Santa Clara, CA, USA). The arrays were then washed and stained with Affymetrix Fluidics Station 450 and scanned on an Affymetrix G7 scanner. All procedures for the RecoverAll kit, FlashTag kit and GeneChip microarrays followed manufacturer’s instructions [18, 19].

The miRNA microarray expression of 847 different human miRNAs was identified and used for statistical analyses.

### Data for validation

#### MiROvaR

To demonstrate the validity of our data we tested the performance of miRNAs included in a recently identified miRNA-based predictor (MiROvaR), of early relapse and progression in OC, on our own dataset [14]. The MiROvaR predictor is based on expression from 35 different human miRNAs, able to stratify patients with high- or low risk of recurrence. The formula for calculation of the prognostic index is briefly described in S1 Appendix, and in previously in details by Bagnoli *et al*.[14]

#### GSE25204, GSE73582 and GSE73581

For validation of our results we retrieved three datasets (GSE25204, GSE73582 and GSE73581) containing clinical information and global miRNA microarray expression profile data. All datasets were retrieved from the NCBI Gene Expression Omnibus database and have been presented and used for validation in previous reports [14, 20].

GSE25204 and GSE73582 were combined and used as one dataset with a total of 263 EOC patients. GSE25204 includes 130 patients with advanced stage (FIGO stage III-IV) EOC [21]. Patients in the GSE73582 cohort amount 133 with FIGO stage I-IV [14]. Microarray expression analysis for the cohorts GSE25204 and GSE73582 were performed on FFPE tissue and frozen samples, and profiled on Illumina miRNA BeadChips Array (Illumina, San Diego, CA, USA).

The GSE73581 cohort includes 179 patients with EOC with FIGO stage I-IV [14, 22]. MiRNA profiling for this cohort was performed on Agilent Microarray Kits (Agilent Technologies, Santa Clara, CA, USA).

Information on OS, TTP and residual disease was available in all external cohorts and defined corresponding to our definitions. Information on PFS was not available in any of the external cohorts. Criteria for tumor cellularity in the tissue samples was more than 70% and necrosis less than 20% in all external cohorts. Detailed information on baseline characteristics and methods for the RNA isolation and microarray analysis in the validation cohorts has been described in detail in the previous studies [14, 21].

Due to comparison between different microarray platforms with different miRNA annotation, all miRNAs identified and described in this study were identified in the miRbase Tracker tool and re-annotated according to the latest miRbase release at the sequence level [23].

### Data processing and statistical analyses

#### Explorative cohort

Initial data analysis was performed in R (R Development Core Team, Vienna, Austria, http://www.R-project.org) using the justRMA function in the Bioconductor package (open source software for bioinformatics) [24]. Intensity measures were background adjusted and normalized by the quantile method with the resulting expression levels on a log2 based scale.

The complete miRNA data files are available from the NCBI’s Gene Expression Omnibus database under the accession number GSE94320.

OS was estimated using the Kaplan-Meier method. Cox proportional hazards models were used for both univariate and multivariate analyses of OS, TTP and PFS. Secondary univariate and multivariate analyses of chemotherapy resistance were performed with logistic regression analysis with chemotherapy resistance binary categorized as resistant or sensitive.

Initially, univariate cox analyses of the log2 expression levels of 847 human miRNAs were performed for all outcomes, and significant miRNAs with a p-value < 0.001 were selected. The multivariate cox analysis was then performed using the selected, significant miRNAs with backwards selection. Final multivariate analyses were verified by ten-fold cross validation to reduce potential overfitting.

The multivariate analyses were after the determination of miRNAs adjusted for histologic subtype, FIGO stage, age, ECOG performance status, body mass index (BMI), and residual tumor after primary surgery. Preliminary analyses of interactions between relevant clinical parameters were performed.

#### Validation

To demonstrate the validity of our data, cox univariate hazard regression model was used to estimate the hazard ratio of the 35 unique miRNAs in our cohort that were included in the MiROvaR predictor (S1 Table).

For validation of our results, miRNAs identified of significance in our univariate (S2 Table) and multivariate (Table 2) cox regression analyses were tested in the two validation cohorts GSE25204+GSE73582 and GSE73581. Due to lack of clinical information in the validation cohorts, the only known prognostic factors included in the multivariate analyses of the GSE25204+GSE73582 cohort were: age, FIGO stage and residual disease, for the GSE73582 cohort histology was also included.

**Table 2:** Multivariate Cox regression analysis of miRNAs associated with PFS, OS and TTP.

	HR	95% CI	p-value
**OS**			
miRNAs			
miR-1183	1.42	1.17–1.74	**0.0005**
miR-126-3p^R^	1.38	1.11–1.71	**0.0036**
Histologic type			
Serous adenocarcinoma	1	-	-
Mucinous adenocarcinoma	1.40	0.58–3.42	0.456
Endometrioid adenocarcinoma	0.46	0.16–1.34	0.157
Clear Cell adenocarcinoma	1.18	0.38–3.64	0.769
FIGO stage			
I	1	-	-
II	4.50	1.51–13.35	**0.0068**
III	5.76	2.23–14.89	**0.0003**
IV	7.17	2.56–20.14	**0.0002**
Age per 10 years	1.24	1.04–1.47	**0.0143**
Performance score^1^ low vs. high	1.25	0.78–1.98	0.354
BMI group			
18.5–25	1	-	-
<18.5	4.42	1.67–11.72	**0.0028**
>25–30	1.24	0.82–1.88	0.305
>30	1.25	0.67–2.34	0.486
Residual tumor			
Yes	1	-	**-**
No	0.41	0.26–0.63	**<0.0001**
**TTP**			
miRNAs			
miR-139-3p^R^	1.48	1.13–1.94	**0.0047**
miR-802^R^	0.48	0.29–0.78	**0.0035**
Histologic type			
Serous adenocarcinoma	1	-	-
Mucinous adenocarcinoma	0.99	0.34–2.91	0.985
Endometrioid adenocarcinoma	0.04	0.01–0.38	**0.0044**
Clear Cell adenocarcinoma	2.27	0.64–8.09	0.206
FIGO stage			
I	1	-	-
II	7.64	1.90–30.81	**0.0042**
III	11.78	3.26–42.59	**0.0002**
IV	9.44	2.39–37.33	**0.0014**
Age per 10 years	1.11	0.94–1.32	0.218
Performance score^1^ low vs. high	1.24	0.74–2.06	0.416
BMI group			
18.5–25	1	-	-
<18.5	2.01	0.56–7.25	0.284
>25–30	1.25	0.81–1.91	0.308
>30	2.50	1.32–4.73	**0.0047**
Residual tumor			
Yes	1	-	-
No	0.33	0.21–0.53	**< 0.0001**
**PFS**			
miRNAs			
miR-23a-5p^R^	1.32	1.09–1.61	**0.0044**
miR-23a-3p^R^	1.70	1.15–2.51	**0.0074**
miR-802^R^	0.48	0.29–0.80	**0.0048**
Histologic type			
Serous adenocarcinoma	-	-	-
Mucinous adenocarcinoma	1.26	0.42–3.77	0.677
Endometrioid adenocarcinoma	0.36	0.08–1.54	0.168
Clear Cell adenocarcinoma	2.78	0.93–8.32	0.067
FIGO stage			
I	1	-	-
II	14.03	3.60–54.67	**0.00014**
III	18.93	5.35–67.00	**<0.00001**
IV	25.32	6.68–96.04	**<0.00001**
Age per 10 years	1.10	0.94–1.28	0.223
Performance score^1^ low vs. high	1.33	0.83–2.13	0.242
BMI group			
18.5–25	1	-	-
<18.5	8.00	2.71–23.64	**0.00017**
>25–30	1.15	0.76–1.73	0.505
>30	1.47	0.81–2.66	0.208
Residual tumor			
Yes	1	-	-
No	0.35	0.22–0.55	**<0.00001**

HR = Hazard ratio, CI = confidence interval, FIGO = International Federation of Gynecology and Obstetrics, OS = overall survival, TTP = time to progression, PFS = progression free survival,

^R^ indicates miRNAs that recur in several analyses

^1^WHO/Eastern Cooperative Oncology Group performance score

In our explorative cohort, statistical significance was defined by a *P-*value < 0.001 for all univariate analyses to compensate for multiple testing in the selection of miRNAs. In the remaining analyses a *P*-value < 0.05 was considered statistical significant. The results are presented as hazard ratios or odds ratios, where applicable with 95% confidence intervals. The discriminatory power of the models, including only the chosen miRNAs, as well as the full multivariable models is assessed using the concordance index (C-Index), i.e. the probability of an event for a randomly selected patient with a higher score is greater than for a patient with a lower score [25]. The statistical analyses were performed using SAS statistical software packaged (version 9.4, Cary N.C. USA), R (v 3.1.0 R Development Core team, Vienna, Austria, http://www.R-project.org), and IBM SPSS statistical software version 19.

### Ethics statements

All patients included in the Pelvic Mass study are informed both orally and in writing, and written informed consent are obtained from all subjects prior to inclusion. The Danish Ethical Committee approved the Pelvic Mass protocol according to the rules of the International Conference on Harmonization/Good Clinical Practice (ICH/GCP) recommendations and the Helsinki and Tokyo conventions (KF01-227/03 and KF01-143/04).

Ethics approval for the validation cohorts has been reported previously [14].

## Results

### Explorative cohort

#### Patients

From the *Pelvic Mass Study*, the first 246 consecutively included patients were identified. A total of 197 patients with EOC fulfilled the inclusion criteria and were included in the study. The remaining 49 patients were excluded according to previous specified exclusion criteria; Non-epithelial OC (n = 2), carcinosarcomas (n = 5), neoadjuvant chemotherapy (n = 15), concomitant cancer (n = 3), insufficient tumor tissue for analysis (n = 24). Clinical and pathologic information on our cohort are summarized in Table 1. Information from the validation cohorts are presented in S3 Table. Patients in our cohort were slightly older at diagnosis compared to the validation cohorts.

#### Progression and survival

Patients were followed from October 2004 until January 17^th^, 2015, five years after the last patient was included, and none were lost to follow-up. All 197 patients were used in the evaluation of PFS and OS. Only the 170 patients who had received chemotherapy were eligible for evaluation of TTP. Median follow-up was 88 months, with the shortest follow-up time for a patient still alive on 61 months. At end of follow-up, 133 (67.5%) patients had died, and 64 (32.5%) patients were still alive. 140 (71.1%) patients had experienced relapse or progressive disease, and 53 (26.9%) patients were alive without signs of relapse or progressive disease, two (1%) died of causes before any signs of progression and two (1%) had died of OC without any information on relapse.

In the univariate analysis 13 miRNAs were identified to correlate with OS, 11 microRNAs were identified to correlate with TTP, and 10 microRNAs were identified to correlate with PFS (p-value < 0.001). Seven miRNAs recurred within PFS and TTP, four miRNAs recurred within PFS and OS and five miRNAs recurred within OS and TTP (S2 Table).

In the multivariate models, two miRNAs (miR-1183, miR-126-3p) remained independent predictors of OS (hazard ratio (HR): 1.42, 95% CI: 1.17–1.74, p-value = 0.0005/ HR: 1.38, 95% CI: 1.11–1.71, p-value = 0.0036), three miRNAs (miR-23a-5p, miR-23a-3p, miR-802) remained independent predictors of PFS (HR: 1.32, 95% CI: 1.09–1.61, p-value = 0.0044/ HR: 1.70, 95% CI: 1.15–2.51, p-value = 0.0074/ HR: 0.48, 95% CI: 0.29–0.80, p-value = 0.0048), and two miRNAs (miR-139-3p, miR-802) remained independent predictors of TTP (HR: 1.48, 95% CI: 1.13–1.94, p-value = 0.0047/ HR: 0.48, 95% CI: 0.29–0.78, p-value = 0.0035) (Table 2).

The discriminatory power of the prognostic miRNAs (concordance index) for all outcomes was 0.76–0.84 (S5 Table)

#### Resistance to chemotherapy

A total of 170 patients received chemotherapy, out of them, 124 (72.9%) were considered chemotherapy sensitive, 26 (15.3%) were considered chemotherapy resistant and 20 (11.8%) were considered chemotherapy refractory. The majority received a combination of Carboplatin and Docetaxel (n = 164, 96.5%), one (0.6%) received Carboplatin and Paclitaxel, and 5 (2.9%) received single agent treatment with Carboplatin (Table 1).

Univariate logistic regression analysis showed that five miRNAs (miR-1234-3p, miR-140-3p, miR-195-5p, miR-223-3p, miR-383-5p) were differentially expressed between chemotherapy sensitive and chemotherapy resistant tumors (p-value < 0.001) (S4 Table) and remained significant after correction for multiple testing. After adjustment for relevant clinical factors, only miR-1234-3p maintained an independent predictive effect (odds ratio: 0.26, 95% CI: 0.11–0.64, p-value = 0.003) (Table 3).

**Table 3:** Multivariate logistic regression analysis for chemotherapy resistance.

	OR	95% CI	P-value
miRNA			
miR-1234-3p	0.26	0.11–0.64	**0.003**
Histologic type			
Serous adenocarcinoma	-	-	-
Mucinous adenocarcinoma	0.27	0.03–2.44	0.244
Endometrioid adenocarcinoma	1.34	0.22–8.16	0.754
Clear Cell adenocarcinoma	5.22	0.72–37.83	0.102
FIGO stage			
I			
II	55.70	0.00–1.88^73^	0.962
III	105.63	0.00–3.54^73^	0.956
IV	184.28	0.00–6.18^73^	0.951
Age per 10 years	1.08	0.73–1.60	0.707
Performance score^1^ low vs. high	0.88	0.49–1.55	0.648
BMI group			
18.5–25	-	-	-
<18.5	968.93	0.00–6.47^155^	0.968
>25–30	0.09	0.00–7.93^49^	0.968
>30	0.18	0.00–1.60^50^	0.977
Residual tumor			
Yes	-	-	-
No	0.45	0.25–0.80	**0.0064**

OR = odds ratio, CI = confidence interval, FIGO = International Federation of Gynecology and

Obstetrics.

^1^WHO/Eastern Cooperative Oncology Group performance score.

### Validation

#### MiROvaR

When miRNAs from the MiROvaR predictor was tested in our cohort, 11 out of 35 miRNAs were identified, and significantly associated with risk of disease progression. Five miRNAs were correlated to an improved prognosis, and six miRNAs correlated to a worse prognosis (S1 Table). The majority of miRNAs included in the MiROvaR predictor, where cross validation support was poor, was also not identified as significant in our cohort (S1 Table). Calculation of the prognostic risk index for MiROvaR with a threshold of 0.07359 was inapplicable due to differences in expression levels across the microarray platforms and resulted in no discrimination of patients. However, if instead the median was calculated, a significant result was obtained.

#### GSE25204, GSE73582 and GSE73581

Out of the 11 miRNAs identified as significant predictors of TTP in our univariate analyses, four miRNAs (miR-139-3p, miR-23a-5p, miR-27a-5p, miR-665) were significantly validated in univariate analysis of the merged cohort GSE25204 + GSE73582, and three (miR-139-3p, miR-23a-5p, miR-483-5p) were significantly validated in the GSE73581 cohort (Tables 4 and 5). None of the 9 miRNAs prognostic for OS in our univariate analysis were significantly validated in any of the external cohorts in univariate analyses (Tables 4 and 5).

**Table 4:** Univariate validation in the GSE25204+GSE73582 cohort.

	HR	95% CI	P-value
**OS**			
**miR-1183**	0.93	0.81–1.07	0.311
**miR-126-3p**	0.85	0.63–1.15	0.288
**miR-198**	0.97	0.88–1.08	0.593
**miR-23a-5p**	0.93	0.70–1.24	0.629
**miR-23a-3p**	0.89	0.69–1.15	0.374
**miR-27a-5p**	1.16	0.95–1.41	0.148
**miR-451a**	0.93	0.78–1.10	0.399
**miR-483-5p**	1.09	0.97–1.23	0.158
**miR-665**	0.94	0.70–1.26	0.673
**TTP**			
**miR-125a-3p**	1.06	0.93–1.21	0.376
**miR-126-3p**	1.18	0.95–1.47	0.129
**miR-138-5p**	1.18	0.99–1.41	0.066
**miR-139-3p**	1.20	1.01–1.43	**0.035**
**miR-23a-5p**	1.35	1.13–1.63	**0.001**
**miR-23a-3p**	1.11	0.92–1.34	0.292
**miR-27a-5p**	1.22	1.04–1.42	**0.012**
**miR-27a-3p**	1.04	0.87–1.25	0.633
**miR-619-3p**	1.04	0.91–1.20	0.540
**miR-665**	1.36	1.10–1.68	**0.004**
**miR-802**	1.01	0.82–1.25	0.923

OS = overall survival, HR = hazard ratio, CI = confidence interval, TTP = time to progression. Significant p-values are marked in bold.

**Table 5:** Univariate validation in GSE73581 cohort.

	HR	95% CI	P-value
**OS**			
**miR-1183**	1.01	0.78–1.33	0.921
**miR-126-3p**	1.13	0.96–1.33	0.135
**miR-198**	0.95	0.73–1.25	0.720
**miR-23a-3p**	1.10	0.92–1.33	0.289
**miR-23a-5p**	1.34	0.93–1.93	0.121
**miR-27a-3p**	1.25	0.98–1.59	0.073
**miR-451a**	1.08	0.96–1.21	0.191
**miR-483-5p**	1.11	0.91–1.36	0.302
**miR-665**	1.02	0.68–1.53	0.914
**TTP**			
**miR-125a-3p**	1.05	0.91–1.22	0.478
**miR-126-3p**	1.01	0.90–1.14	0.832
**miR-139-3p**	1.41	1.05–1.90	**0.022**
**miR-23a-3p**	0.98	0.86–1.12	0.799
**miR-23a-5p**	1.58	1.18–2.12	**0.002**
**miR-27a-3p**	1.07	0.90–1.27	0.466
**miR-665**	1.26	0.93–1.71	0.134

OS = overall survival, HR = hazard ratio, CI = confidence interval, TTP = time to progression. Significant p-values are marked in bold.

Of the miRNAs prognostic for OS (miR-1183, miR-126) and TTP (miR-139-3p, miR-802) in our multivariate analysis (Table 2), only miR-139-3p was significantly validated in univariate analysis of one of the external cohorts (GSE25204+GSE73582) (Table 4). miR-1183 showed significant correlation to OS in multivariate validation, but with an adverse outcome (S6 Table). miR-802, miR-138-5p, and miR-619-3p were not included in the Agilent miRNA microarray, used for miRNA profiling in the GSE73581 cohort, and therefore not possible to validate in this cohort (S7 Table).

## Discussion

Unfortunately, most OC patients are diagnosed in late stages, where the disease has already spread beyond the ovaries. Despite a relatively good response to first line chemotherapy and adequate primary surgery, the majority of patients eventually develop resistance leading to relapse and treatment failure [26]. Therefore, methods that can identify OC patients with a poor prognosis and high risk of chemotherapy resistance are urgently needed to improve individualized treatment. Several studies have shown that miRNAs have a potential as novel predictive markers of prognosis and progression in cancer [27–31]. However, diverging results calls for further research and validation of existing data with new more reliable technologies.

In the current study we identified specific miRNAs that showed significant association with OS, PFS, TTP and resistance to chemotherapy in our explorative cohort of patients with EOC. The validity of our data was demonstrated by significant performance of a handful of miRNAs from a previous developed miRNA-based predictor of early relapse and progression in OC (MiROvaR). Validation of our results was attempted on three public accessible datasets, and a few miRNAs were significantly validated in univariate analysis of the external cohorts.

In our primary statistical analyses miR-1183 and miR-126-3p retained its prognostic effect of OS, miR-139-3p and miR-802 of TTP, and miR-23a-3p, miR-23a-5p and miR-802 of PFS, in multivariate analysis. In multivariate analysis of chemotherapy resistance miR-1234 was found significantly correlated to improved response to chemotherapy. A promising discriminatory power of the miRNAs was shown. A conservative choice for selection of statistical significance is necessary in order to avoid false positive choices, and results in the limited number of miRNAs that are likely associated with the clinical outcomes.

In the validation of the prognostic miRNAs found in multivariate analysis of our explorative cohort, only miR-139-3p, was significantly validated in univariate analysis of both external cohorts. However, a few other miRNAs from our univariate analyses of TTP were significantly validated, but only miR-23a-5p recurred in both external cohorts. None of the miRNAs identified as prognostic for OS were validated.

Out of the identified miRNAs of significance, miR-1183 has not previously been described in association with OC, and reporting of its association in other diseases is very limited, however, one study on rectal cancer, found miR-1183 to be upregulated in patients with locally advanced disease and associated with a complete response to neoadjuvant chemotherapy [32]. miR-126 has been described with altered expression in cancer, and to act both as an oncogene and tumor suppressor. Only one previous study was found in the literature describing the function of miR-126 in relation to OC, and here it was described to act as a tumor suppressor in OC cell lines, possibly by suppression of the serine/threonine-protein kinase PAK4, an enzyme known to increase cellular motility, invasion, metastasis, and growth [33]. This is contradicting our results, where higher expression of miR-126 was associated with shorter OS. On the other hand, miR-23a has also been described to act as an oncogene in different cancers, including OC, where it was found to increase cell growth and inhibit apoptosis in OC cell lines [34–36]. A recent study also found miR-23a negatively correlated with survival, tumor differentiation, lymph node metastasis and clinical staging in OC [37], supporting our findings, where higher miR-23a expression was correlated to shorter OS and PFS. miR-802 has in several previous reports been described to act as a tumor suppressor; in breast cancer miR-802 was found down regulated, and *in vivo* and *in vitro* upregulation of miR-802 showed to suppress the cancer growth by down regulation of the oncogene FoxM1 [38]. In gastric cancer miR-802 was down regulated and found to act as a tumor suppressor by directly targeting RAB23 [39]. Likewise, in tongue and prostate cancer, miR-802 has been described with a tumor suppressing function [40, 41]. In prostate cancer miR-802 was found to suppress epithelial-mesenchymal transition (EMT) [40]. All these studies are in line with, and support our results, where increased expression of miR-802 was found to be significantly associated with longer PFS and TTP. miR-139-3p has primarily been described with tumor suppressing functions, contradicting our results, where we find it associated with shorter TTP [42, 43]. However, one study found miR-139-3p upregulated in colorectal cancer with liver metastasis, and potentially associated with poor survival, in agreement with our results [44].

Despite the initial good response to platinum-based chemotherapy, resistance develops in the majority of OC patients and acquired resistance, is as for other cancers one of the major reasons for a poor survival. The molecular reasons for acquired resistance, have been thoroughly investigated, and are usually a combination of different mechanisms, that overall can be categorized as pre-, on-, post-, or off target mechanisms [45]. Pre-target mechanisms prevent accumulation of the drug in the cell by efflux to the extracellular environment. In on-target mechanisms DNA damage are repaired more efficiently. Post-target mechanisms prevent apoptosis of the cancer cells and off-target mechanisms comprise mechanisms that do not directly interact with the platinum-based drug, but can compensate for the effects thus neutralizing the actions of the drug. Several studies of OC have demonstrated that miRNAs may affect these mechanisms and could be potential targets for overcoming resistance to chemotherapy [45, 46]. However, no studies on miR-1234 in relation to chemotherapy resistance were found in the literature, but a few studies on different cancer types were found. In B-cell lymphoma miR-1234 was found to repress expression of STAT3 protein an oncogenic transcription factor [47], and in nasopharyngeal carcinoma found to be positively associated with OS [48]. We found miR-1234 to be positively associated with response to chemotherapy; higher expression level was correlated with improved response to chemotherapy.

Validation of miR-1234 in the external cohorts was not possible due to lack of clinical information on chemotherapy and resistance to chemotherapy. Nonetheless, identification of patients that might be resistant to chemotherapy would potentially allow for more aggressive follow-up of this subgroup, and earlier shift to second line chemotherapeutics, which hopefully would result in an overall improved survival for the patients. Therefore, further functional studies of miR-1234 that could elucidate the regulatory pathways of the miRNA and validation in a prospective cohort study with sufficient power would be of great interest.

The weaknesses of our study, worth notifying, and elements that could explain the inconsistency from previous studies are, that we only included patients from one Danish center, and miRNA was profiled with Affymetrix miRNA microarrays, but validation was performed in cohorts analyzed with two different array platforms (Agilent- and Illumina miRNA microarrays). The results were not validated with qRT-PCR, which could be addressed as a limitation. However, validation in external cohorts must be considered as a more reliable form of validation limiting the risk of repeating false results. Further, miRNA microarray expression has shown to be highly concordant when re-analyzed with qRT-PCR [49, 50], with correlation coefficients measuring from r = 0.986 to 0.994, depending on normalization method [51].

An important strength of the current study is the mature follow-up data, where the shortest follow-up time was 61 months for a patient still alive. Also, the cohort consisted of a consecutive inclusion of patients, without restriction regarding socio-economic status of patients referred to the clinic. Data was collected prospectively, independent of outcomes, and continuously updated in the Danish Gynecologic Database. None were lost to follow-up, and missing information was minimal. The size of our cohort was comparable to the two validation cohorts. However, besides the longer follow-up time, patients were slightly older at diagnosis, less patients had advance stage disease and more patients obtained complete cytoreductive surgery, in our cohort compared to the validation cohorts (Table 1 and S2 Table).

Validation of the MiROvaR predictor on our cohort showed that 11 miRNAs were identified as significantly, associated with PFS. The majority of miRNAs that showed poor cross validation in the study by Bagnoli et al. were expectedly not identified as significant in our study. Of the significant miRNAs associated with a good prognosis miR-506, miR-508 and miR-509 are miRNAs previously described and known to act as tumor-suppressors in OC, supporting that our study cohort are representative and proven with the used technology [52–54].

A major problem and a reason for lack of validation and discordance between identified prognostic miRNAs could be attributed to inconsistency in RNA purification methods, data management, the used microarray technology, intra-laboratory variance, besides batch effects with day-to-day variations in measurements [55]. Results of intra-platform correlation and data comparison have been reported with diverging conclusions in several studies [56–59]. In our study, our primary results were performed on Affymetrix miRNA microarray platforms where the miRNA expression of 847 different human miRNAs were analyzed, validation of our results were performed on two different datasets using Illumina and Agilent miRNA microarray platforms. It has been shown that there might be better concordance of results between Illumina and Agilent platforms, while Affymetrix might show less reliability and agreement with the other platforms [60]. Preferably, the results should have been validated on data using the same platform. Unfortunately, no other global miRNA microarray studies of EOC performed on Affymetrix microRNA microarray GeneChip were available from the Gene Expression Omnibus database [20].

Various commercialized microarray platforms exist today, and the lack of standardization in platform fabrication, and analysis methods challenge the comparison of results of different investigations, and implicates the usefulness of existing, experimental material. However, at least some approaches have been made to facilitate the exchange and analysis of data. The Minimum Information About a Microarray Experiment (MIAME) is a project that has developed guidelines for the required information that needs to be provided to enable public available microarray-based data for interpretation and independent verification [61]. And the FDA has developed the MicroArray Quality Control (MAQC) to establish standards and quality measures for microarray and next-generation sequences technologies to ensure successful and reliable methods that can be used in clinical practice, and regulatory decision-making [62].

## Conclusion

In the current study predictors of survival and resistance to chemotherapy were developed based on global miRNA expression profiles from tumor tissue of EOC patients. A number of different miRNAs were shown to act as significant and independent predictors of OS, TTP and PFS; and one miRNA showed prominent differentiation between patients who were chemotherapy sensitive and patients who were chemotherapy resistant. A few of the identified miRNAs were possible to validate in external cohorts (miR-23a-5p and miR-139-3p) and may be of importance for identifying patients at risk of early relapse.Further functional studies that can elucidate the regulatory pathways of the identified miRNAs would be of great interest, and validation of our prognostic and predictive results in large prospective cohorts are warranted. However, a major challenge exist in comparison of miRNA microarray data across studies using different platforms, and techniques due to lack of standardized methods for platform fabrication, assay protocols and data processing [63]. Validation of the prognostic and predictive results should therefore be performed in cohorts where the miRNA profiling is performed on Affymetrix microarray platforms.

## Supporting information

S1 Appendix MiROvaR. (DOCX)

S1 Table Comparison of miRNAs used in the MiROvaR predictor and our explorative cohort. (DOCX)

S2 Table Univariate Cox regression analysis of miRNAs associated with PFS, OS and time-to-progression. (DOCX)

S3 Table Baseline characteristics of validation cohorts. (DOCX)

S4 Table Univariate logistic regression analysis of miRNAs associated with chemotherapy resistance. (DOCX)

S5 Table Concordance index. (DOCX)

S6 Table Multivariate validation in the GSE25204+GSE73582 cohort. (DOCX)

S7 Table Multivariate validation in the GSE73581 cohort. (DOCX)

S1 Clinical Data (SAV)
