# Supplementary material for: Identification and validation of potential prognostic and predictive miRNAs of epithelial ovarian cancer
Source: PLoS One. 2018 Nov 26;13(11):e0207319. doi: 10.1371/journal.pone.0207319 (PMC6261038; doi:10.1371/journal.pone.0207319)
Supplement: S1 Appendix — (DOCX) [file pone.0207319.s001.docx]

**S1 Appendix. MiROvaR**

Shortly, the molecular MiROvaR predictor was developed with cox proportional hazards regression analysis of miRNAs associated with PFS and a principal component analysis to reduce the dimensionality of all the miRNAs selected for the model. The prognostic index for the individual patients was calculated with the following formula:

∑_i_*w_i_ x_i_ +* 3 ⋅ 196617

Where *w_i_* is the weight and *x_i_* is the logarithmically transformed miRNA expression of the *i*-th miRNA intensity. The threshold for the prognostic index was set to 0.07359 corresponding to the median value obtained in a 10-fold cross validation, which classifies the patients as being at high or low risk of progression or relapse. An index value above the threshold was predicted as high risk of progression or relapse and an index below the threshold as low risk of progression or relapse.
