# Supplementary material for: Identification and validation of potential prognostic and predictive miRNAs of epithelial ovarian cancer
Source: PLoS One. 2018 Nov 26;13(11):e0207319. doi: 10.1371/journal.pone.0207319 (PMC6261038; doi:10.1371/journal.pone.0207319)
Supplement: S1 Table — (DOCX) [file pone.0207319.s002.docx]

| **S1 Table. Comparison of miRNAs used in the MiROvaR predictor and our explorative cohort** | | | | | | | | |
| --- | --- | --- | --- | --- | --- | --- | --- | --- |
|  | **Validation in our cohort** | | |  | **MiROvaR** | | | |
|  | **HR** | **95% CI** | **P-value** |  | **HR** | **95% CI** | **P-value** | **Cross validation support** |
| **miR-141-3p** | 1.05 | 0.92 - 1.20 | 0.4737 |  | 0.82 | 0.73 - 0.92 | 0.0019 | 100% |
| **miR-200a-3p** | 1.05 | 0.93 - 1.18 | 0.4325 |  | 0.81 | 0.71 - 0.93 | 0.0032 | 100% |
| **miR-200b-3p** | 1.04 | 0.92 - 1.19 | 0.5230 |  | 0.79 | 0.68 - 0.91 | 0.0027 | 100% |
| **miR-200c-3p** | 1.07 | 0.88 - 1.29 | 0.5137 |  | 0.79 | 0.69 - 0.91 | 0.0015 | 100% |
| **miR-506-3p** | 0.80 | 0.71 - 0.90 | 0.0001 |  | 0.64 | 0.48 - 0.84 | 0.0001 | 100% |
| **miR-507** | 0.62 | 0.35 - 1.10 | 0.1032 |  | 0.59 | 0.43 - 0.81 | 0.0001 | 100% |
| **miR-508-3p** | 0.81 | 0.69 - 0.95 | 0.0104 |  | 0.75 | 0.64 - 0.87 | 0.0001 | 100% |
| **miR-509-3p** | 0.92 | 0.87 - 0.96 | 0.0005 |  | 0.78 | 0.69 - 0.90 | 0.0001 | 100% |
| **miR-509-5p** | 0.82 | 0.74 - 0.92 | 0.0003 |  | 0.68 | 0.56 - 0.84 | 0.0001 | 100% |
| **miR-513a-5p** | 0.95 | 0.87 - 1.04 | 0.2413 |  | 0.77 | 0.66 - 0.89 | 0.0007 | 100% |
| **miR-513b-5p** | 0.85 | 0.73 - 0.99 | 0.0330 |  | 0.82 | 0.73 - 0.91 | 0.0007 | 100% |
| **miR-514a-3p** | 0.91 | 0.59 - 1.41 | 0.6788 |  | 0.81 | 0.73 - 0.91 | 0.0001 | 100% |
| **miR-592** | 0.74 | 0.43 - 1.28 | 0.2819 |  | 0.26 | 0.10 - 0.66 | 0.0002 | 100% |
| **miR-135b-5p** | 0.66 | 0.37 - 1.16 | 0.1458 |  | 0.85 | 0.76 - 0.96 | 0.0089 | 80% |
| **miR-429** | 0.93 | 0.66 - 1.32 | 0.6922 |  | 0.84 | 0.73 - 0.96 | 0.0122 | 60% |
| **miR-890** | 1.01 | 0.80 - 1.28 | 0.9150 |  | 0.09 | 0.01 - 0.72 | 0.0231 | 40% |
| **miR-29c-5p** | 1.07 | 0.84 - 1.36 | 0.5746 |  | 1.60 | 1.23 - 2.07 | 0.0007 | 100% |
| **miR-193a-5p** | 1.35 | 1.09 - 1.66 | 0.0051 |  | 1.98 | 1.49 - 2.61 | 0.0001 | 100% |
| **miR-30b-3p** | 1.02 | 0.86 - 1.20 | 0.8517 |  | 1.98 | 1.24 - 3.17 | 0.0064 | 100% |
| **miR-486-5p** | 1.12 | 1.03 - 1.23 | 0.0106 |  | 1.35 | 1.12 - 1.61 | 0.0030 | 90% |
| **miR-423-5p** | 1.00 | 0.85 - 1.18 | 0.9826 |  | 1.77 | 1.23 - 2.54 | 0.0029 | 90% |
| **miR-100-3p** | 0.79 | 0.48 - 1.32 | 0.3761 |  | 1.96 | 1.29 - 2.97 | 0.0090 | 90% |
| **miR-484** | 1.12 | 0.91 - 1.38 | 0.2726 |  | 1.60 | 1.16 - 2.21 | 0.0079 | 80% |
| **miR-23a-5p** | 1.62 | 1.36 - 1.94 | <0.0001 |  | 1.64 | 1.18 - 2.28 | 0.0052 | 80% |
| **miR-143-5p** | 1.01 | 0.89 - 1.15 | 0.8595 |  | 1.67 | 1.18 - 2.37 | 0.0096 | 80% |
| **miR-330-3p** | 0.97 | 0.84 - 1.12 | 0.6869 |  | 1.86 | 1.21 - 2.85 | 0.0061 | 80% |
| **miR-99b-5p** | 1.37 | 1.10 - 1.70 | 0.0046 |  | 1.35 | 1.08 - 1.70 | 0.0094 | 70% |
| **miR-769-5p** | 1.00 | 0.84 - 1.19 | 0.9913 |  | 1.76 | 1.19 - 2.61 | 0.0082 | 70% |
| **miR-452-5p** | 1.27 | 1.08 - 1.51 | 0.0049 |  | 1.28 | 1.06 - 1.53 | 0.0175 | 60% |
| **miR-151a-3p** | 1.06 | 0.89 - 1.27 | 0.5069 |  | 1.36 | 1.03 - 1.51 | 0.0134 | 60% |
| **miR-193b-5p** | 1.30 | 1.08 - 1.56 | 0.0050 |  | 1.51 | 1.06 - 2.14 | 0.0241 | 60% |
| **miR-574-5p** | 0.87 | 0.72 - 1.04 | 0.1159 |  | 1.28 | 1.05 - 1.57 | 0.0161 | 50% |
| **miR-29a-5p** | 0.81 | 0.46 - 1.42 | 0.4583 |  | 1.77 | 1.15 - 2.70 | 0.0179 | 50% |
| **miR-30d-5p** | 1.08 | 0.90 - 1.29 | 0.4068 |  | 1.25 | 1.03 - 1.52 | 0.0233 | 40% |
| **miR-195-3p** | 1.01 | 0.77 - 1.32 | 0.9281 |  | 1.63 | 1.13 - 2.36 | 0.0187 | 40% |

HR = hazard ratio, CI = confidence interval

Green indicates significant miRNAs associated with a good prognosis.

Red indicates significant miRNAs associated with a poor prognosis.
