# Supplementary material for: Identification and validation of potential prognostic and predictive miRNAs of epithelial ovarian cancer
Source: PLoS One. 2018 Nov 26;13(11):e0207319. doi: 10.1371/journal.pone.0207319 (PMC6261038; doi:10.1371/journal.pone.0207319)
Supplement: S2 Table — (DOCX) [file pone.0207319.s003.docx]

| **S2 Table. Univariate Cox regression analysis of miRNAs associated with PFS, OS and time-to-progression.** | | |
| --- | --- | --- |
|  | **HR** | **p-value** |
| **OS** |  |  |
| hsa-miR-1183 | 1.38 | 0.0003 |
| hsa-miR-126-3p^R^ | 1.44 | 0.0002 |
| hsa-miR-198 | 1.39 | 0.0002 |
| hsa-miR-23a-5p^R^ | 1.47 | <0.0001 |
| hsa-miR-23a-3p^R^ | 2.04 | 0.0001 |
| hsa-miR-27a-5p^R^ | 1.37 | 0.0002 |
| hsa-miR-451a | 1.28 | 0.0003 |
| hsa-miR-483-5p^R^ | 1.19 | 0.0007 |
| hsa-miR-665^R^ | 1.63 | <0.0001 |
| **TTP** |  |  |
| hsa-miR-125a-3p^R^ | 1.47 | 0.0006 |
| hsa-miR-126-3p^R^ | 1.40 | 0.0009 |
| hsa-miR-138-5p^R^ | 1.44 | <0.0001 |
| hsa-miR-139-3p^R^ | 1.46 | <0.0001 |
| hsa-miR-23a-5p^R^ | 1.51 | <0.0001 |
| hsa-miR-23a-3p^R^ | 1.96 | 0.0002 |
| hsa-miR-27a-5p^R^ | 1.42 | <0.0001 |
| hsa-miR-27a-3p | 1.50 | 0.0003 |
| hsa-miR-619-3p | 0.46 | 0.0009 |
| hsa-miR-665^R^ | 1.58 | 0.0002 |
| hsa-miR-802^R^ | 0.39 | <0.0001 |
| **PFS** |  |  |
| hsa-miR-125a-3p^R^ | 1.54 | 0.0002 |
| hsa-miR-138-5p^R^ | 1.36 | 0.0003 |
| hsa-miR-139-3p^R^ | 1.41 | 0.0009 |
| hsa-miR-23a-5p^R^ | 1.59 | <0.0001 |
| hsa-miR-23a-3p^R^ | 2.22 | <0.0001 |
| hsa-miR-27a-5p^R^ | 1.48 | <0.0001 |
| hsa-miR-483-5p^R^ | 1.19 | 0.0007 |
| hsa-miR-576-5p | 0.33 | 0.0007 |
| hsa-miR-802^R^ | 0.46 | 0.0004 |

HR = Hazard ratio, OS = overall survival, TTP = time to progression, PFS = progression free survival

^R^ indicates miRNAs that recur in several analyses.
