# Supplementary material for: Identification and validation of potential prognostic and predictive miRNAs of epithelial ovarian cancer
Source: PLoS One. 2018 Nov 26;13(11):e0207319. doi: 10.1371/journal.pone.0207319 (PMC6261038; doi:10.1371/journal.pone.0207319)
Supplement: S4 Table — (DOCX) [file pone.0207319.s005.docx]

| **S4 Table. Univariate logistic regression analysis of miRNAs associated with chemotherapy resistance** | | |
| --- | --- | --- |
| **miRNA** | **OR** | **p-value** |
| miR-1234 | 0.28 | 0.0003 |
| miR-140-3p | 1.9. | 0.0008 |
| miR-195-5p | 1.81 | 0.0007 |
| miR-223-3p | 1.95 | 0.0007 |
| miR-383-5p | 1.72 | 0.0003 |

OR = odds ratio
