# Supplementary material for: Identification and validation of potential prognostic and predictive miRNAs of epithelial ovarian cancer
Source: PLoS One. 2018 Nov 26;13(11):e0207319. doi: 10.1371/journal.pone.0207319 (PMC6261038; doi:10.1371/journal.pone.0207319)
Supplement: S5 Table — (DOCX) [file pone.0207319.s006.docx]

| **S5 Table. Concordance Index.** | | |
| --- | --- | --- |
|  | **Univariate (only miRNAs)** | **Multivariate** |
| **OS** | 0.63 | 0.77 |
| **TTP** | 0.62 | 0.76 |
| **PFS** | 0.68 | 0.77 |
| **Chemotherapy resistance** | 0.67 | 0.84 |

OS= overall survival, TTP= time to progression, PFS= progression free survival.

The concordance index indicates the discriminatory power of the prognostic miRNAs of the given outcome. The following miRNAs were in the calculation of the indexes; miR-1183, miR-126-3p (OS), miR-139-3p, miR-802 (TTP), miR-23a-3p, miR-23a-5p, miR-802 (PFS), miR-1234-3p (chemotherapy resistance).
