# Supplementary material for: Identification and validation of potential prognostic and predictive miRNAs of epithelial ovarian cancer
Source: PLoS One. 2018 Nov 26;13(11):e0207319. doi: 10.1371/journal.pone.0207319 (PMC6261038; doi:10.1371/journal.pone.0207319)
Supplement: S6 Table — (DOCX) [file pone.0207319.s007.docx]

| **S6 Table. Multivariate validation in the GSE25204+GSE73582 cohort** | | | |
| --- | --- | --- | --- |
|  | **HR** | **95% CI** | **P-value** |
| **OS** | | | |
| **miR-1183** | 0.70 | 0.50 - 0.97 | **0.0328** |
| **miR-126-3p** | 0.99 | 0.80 - 1.21 | 0.8950 |
| Age per 10 years | 1.11 | 0.93 - 1.34 | 0.2483 |
| Residual disease |  |  |  |
| NED | - | - | - |
| GRD | 3.96 | 2.11 - 7.45 | **<0.0001** |
| mRD | 2.63 | 1.37 - 5.05 | **0.0035** |
| FIGO stage |  |  |  |
| I | 0.00 | 0.00 - | 0.9806 |
| II | 0.00 | 0.00 - | 0.9890 |
| III | 0.90 | 0.48 - 1.67 | 0.7408 |
| IV | - | - | - |
| **TTP** | | | |
| **miR-139-3p** | 1.05 | 0.97 - 1.14 | 0.2330 |
| **miR-802** | 0.87 | 0.68 - 1.10 | 0.2422 |
| Age per 10 years | 1.07 | 0.94 - 1.22 | 0.2831 |
| Residual disease |  |  |  |
| NED | - | - | - |
| GRD | 1.98 | 1.27 - 3.09 | **0.0026** |
| mRD | 2.11 | 1.33 - 3.35 | **0.0016** |
| FIGO stage |  |  |  |
| I | 0.52 | 0.21 - 1.29 | 0.1557 |
| II | 0.99 | 0.31 - 3.14 | 0.9831 |
| III | 0.99 | 0.60 - 1.62 | 0.9557 |
| IV | - | - | - |

TTP = time to progression, HR = hazard ratio, CI = confidence interval, OS = overall survival, NED = no evident disease, mRD = minimal residual disease (tumor < 1cm), GRD = gross residual disease (tumor >1cm).

* miRNAs identified as prognostic for PFS in our explorative cohort, tested against their TTP since information on PFS was lacking in the external cohorts.

Significant p-values are marked in bold.
