# Supplementary material for: Identification and validation of potential prognostic and predictive miRNAs of epithelial ovarian cancer
Source: PLoS One. 2018 Nov 26;13(11):e0207319. doi: 10.1371/journal.pone.0207319 (PMC6261038; doi:10.1371/journal.pone.0207319)
Supplement: S7 Table — (DOCX) [file pone.0207319.s008.docx]

| **S7 Table. Multivariate validation in the GSE73581 cohort** | | | |
| --- | --- | --- | --- |
|  | **HR** | **95% CI** | **P-value** |
| **OS** | | | |
| **miR-1183** | 1.20 | 0.95 - 1.51 | 0.1350 |
| **miR-126-3p** | 1.14 | 0.84 - 1.56 | 0.3965 |
| Age per 10 years | 1.23 | 0.96 - 1.57 | 0.1019 |
| Histology |  |  |  |
| 2 | - | - | - |
| 3 | 0.83 | 0.37 - 1.85 | 0.6489 |
| 4 | 0.79 | 0.24 - 2.61 | 0.6953 |
| 5 | 4.95 | 1.61 - 15.21 | **0.0052** |
| 6 | 1.27 | 0.47 - 3.42 | 0.6345 |
| Residual disease |  |  |  |
| NED | 8.22 | 3.39 - 19.95 | **<0.0001** |
| mRD | 2.42 | 1.18 - 4.98 | **0.0161** |
| GRD | 2.68 | 1.28 - 5.60 | **0.0088** |
| FIGO stage |  |  |  |
| I | - | - | - |
| II | 4.87 | 0.47 - 50.39 | 0.1840 |
| III | 7.18 | 0.88 - 58.70 | 0.0658 |
| IV | 8.09 | 0.92 - 71.34 | 0.0597 |
| **TTP** | | | |
| **miR-139-3p** | 0.91 | 0.78 - 1.06 | 0.2408 |
| Age per 10 years | 1.04 | 0.87 - 1.25 | 0.6825 |
| Histology |  |  |  |
| 1 | 0.82 | 0.41 - 1.63 | 0.5775 |
| 2 | - | - | - |
| 3 | 0.82 | 0.35 - 1.89 | 0.6348 |
| 4 | 0.77 | 0.27 - 2.18 | 0.6292 |
| 5 | 1.50 | 0.45 - 5.01 | 0.5138 |
| Residual disease |  |  |  |
| NED | 5.47 | 2.58 - 11.62 | **<0.0001** |
| mRD | 2.32 | 1.36 - 3.95 | **0.0019** |
| GRD | 1.82 | 1.09 - 3.06 | **0.0231** |
| FIGO stage |  |  |  |
| I | - | - | - |
| II | 1.20 | 0.31 - 4.62 | 0.7861 |
| III | 3.06 | 1.06 - 8.82 | **0.0386** |
| IV | 4.06 | 1.28 - 12.89 | **0.0174** |

TTP = time to progression, HR = hazard ratio, CI = confidence interval, OS = overall survival, NED = no evident disease, mRD = minimal residual disease (tumor < 1cm), GRD = gross residual disease (tumor >1cm).

* miRNAs identified as prognostic for PFS in our explorative cohort, tested against their TTP since information on PFS was lacking in the external cohorts.

Significant p-values are marked in bold.
